# Construction of a mycelium sphere using a *Fusarium* strain isolate and *Chlorella* sp. for polyacrylamide biodegradation and inorganic carbon fixation

**DOI:** 10.3389/fmicb.2023.1270658

**Published:** 2023-10-05

**Authors:** Huichao Zhang, Mohan Shangguan, Chang Zhou, Zhaoyang Peng, Zhongyi An

**Affiliations:** ^1^School of Civil Engineering, Yantai University, Yantai, China; ^2^The Architectural Design and Research Institute of HIT Co., Ltd., Harbin, China

**Keywords:** *Fusarium* sp., *Chlorella* sp., mycelial pellets, fungal-microalgal consortium, polyacrylamide removal

## Abstract

In the context of global demand for carbon reduction, the formation of inorganic carbon (IC) in the wastewater from oil flooding becomes a potential threat. In this study, *Chlorella* sp. and *Fusarium* sp. were used to assemble a fungal-algal pellet to degrade polyacrylamide (PAM) and fix IC in synthetic oil-flooding wastewater. The results showed that the combination of *Chlorella* sp. and *Fusarium* sp. was more effective at degrading PAM and removing carbon than a monoculture. With PAM as the sole nitrogen source, the degradation of PAM by the consortium was enhanced up to 35.17 ± 0.86% and 21.63 ± 2.23% compared with the monocultures of fungi or microalgae, respectively. The degradation of the consortium was significantly enhanced by the addition of an external nitrogen source by up to 27.17 ± 2.27% and 22.86 ± 2.4% compared with the monoculture of fungi or microalgae, respectively. This may depend on the effect of synergy between the two species. For the removal of IC from the water, the removal efficiency of the consortium was higher than that of the microalgae by 38.5 ± 0.08%, which may be attributed to the ability of the fungi to aid in the adsorption of nutrients and its assimilation by the microalgae. Therefore, the *Fusarium*-*Chlorella* consortium can effectively degrade PAM, while simultaneously fixing carbon, which provides a feasible scheme for the treatment and carbon neutralization of the wastewater that contains PAM.

## 1. Introduction

Polyacrylamide (PAM) is primarily used in the process of alkali/polymer/surfactant flooding, which results in a large amount of wastewater that contains a concentration of 300–800 mg/L PAM (Song et al., 2021). PAM can be hydrolyzed to form toxic acrylamide monomers, which have been shown to damage the nervous system of humans and animals (Albalawi et al., 2018). Furthermore, the carbon dioxide (CO_2_) in the formation is dissolved into the flooding water to form dissolved inorganic carbon (DIC) with a CO_2_ equivalent of 3 to 4 kg/m^3^. The excessive emissions of CO_2_ that cause the greenhouse effect have attracted global attention (Modak et al., 2018). Oversaturated CO_2_ in the water poses the risk of escaping into the atmosphere, thus, affecting the carbon cycle and disrupting the balance of carbon (Giménez-Gómez et al., 2023). Therefore, it is highly necessary to remove the PAM and DIC before the flooding wastewater is discharged.

The current research hotspots on the biodegradation of PAM primarily include two aspects. The first is the use of anaerobic, aerobic or anaerobic-aerobic combination bioreactors to treat PAM, such as sequencing batch reactor (SBR) systems, alkaline pre-fermentation for anaerobic digestion and anaerobic digestion under mesophilic conditions (Liu et al., 2021). Secondly, whether the bacterial strains are used individually or in combination to degrade PAM. In terms of its molecular structure, PAM is composed of main carbon chains and side chain amide groups. The mineralization of PAM requires the degradation of both the main carbon chain and side chain amide groups. Amidases can help microorganisms to degrade amide groups by catalyzing the hydrolysis of amide bonds to form ammonia and carboxylic acids, and these enzymes are produced by a variety of microorganisms, including bacteria, actinomycetes, fungi, and algae. Numerous studies have investigated the biodegradation of PAM by bacterial strains, such as *Bacillus* sp. (Nyyssölä and Ahlgren, 2019), *Acinetobacter* sp. (Zhang et al., 2021) and *Clostridium* sp. (Ma et al., 2010), but to our knowledge, the feasibility of the degradation of PAM by single algae species has not been reported. Similarly, previous studies have shown that the long carbon chains of PAM can be degraded by bacteria. Although there have been few studies on the degradation of long carbon chains of PAM by fungi, these microbes have a strong ability to degrade other complex carbon compounds, including crude oil and polycyclic aromatic hydrocarbons (PAHs) (Zain Ul Arifeen et al., 2022; Saravanan et al., 2023).

Currently, the pollutants in oil flooding wastewater are not limited to PAM but also include soluble inorganic carbon (IC). In previous studies, microalgae have been shown to have a superior ability to remove DIC. Both small and large algae can utilize carbonic anhydrase (CA) to metabolize IC (Vyrides et al., 2018; Thanigaivel et al., 2022). Furthermore, several microalgal species have been found to secrete amidases, including *Chlorella vulgaris, Scenedesmus obliquus, Chlamydomonas reinhardtii, Dunaliella salina,* and *Nannochloropsis oceanica* (Sheng et al., 2022). A fungal-algal particle system is an emerging microbial water treatment technology in which mycelial particles are used as special biological carriers to load algae and then used as microbial materials to treat water. Due to the high efficiency and safety of microbial technology, as well as the resistance to impact and stability of biological materials, it is gradually attracting the attention of researchers (Du et al., 2018; Ma et al., 2021; Li et al., 2023). Many researchers have also proven that fungal-algal pellets are highly efficient at treating organic wastewater (Zhou et al., 2018; Chen et al., 2020). However, few studies have investigated the removal of PAM-containing wastewater using fungi either alone or in a fungal-microalgal consortium.

In this study, *Fusarium* sp. was obtained by screening strains. Subsequently, *Fusarium* sp. and *Chlorella* sp. were co-cultured to form a *Fusarium*-*Chlorella* consortium. Based on the advantages of microalgae in wastewater treatment described above, this study investigated the differences in the efficiency of pollutant degradation among three different biological systems: single *Fusarium* sp. mycelial pellets, single *Chlorella* sp. and the *Fusarium*-*Chlorella* consortium in treating synthetic wastewater, as well as whether the addition of nitrogen sources for co-metabolism could enhance the ability of the three biological systems to degrade organic wastewater.

## 2. Materials and methods

### 2.1. Preparation of the microbial strains

#### 2.1.1. Culturing of the microalgae

The microalgal species used in this study was *Chlorella sorokiniana* (FACHB-26), which was supplied by the Freshwater Algae Culture Collection at the Institute of Hydrobiology, Chinese Academy of Science (FACHB, Wuhan, China). Sterile Bold’s Basal Medium (BBM) (Zhang et al., 2021) supplemented with PAM was used in this study. The species was radiantly acclimatized on a shaker (25 ± 2°C, light intensity of 3,000 l×, 12:12 h light: dark cycle). The initial concentration of PAM was set at 100 mg/L, and the concentration was gradually increased to 400 mg/L during a 20 days acclimatization period. This enabled the species to gradually adapt to the presence of PAM for further experiments.

#### 2.1.2. Isolation and screening of the fungi

The samples used to isolate the filamentous fungi were collected from a revolving algal biofilm (RAB) reactor used to treat the wastewater that contained PAM (Zhang et al., 2021), which had been operating in the laboratory for 1 year. The algal biofilm was collected from the rotary belt of the RAB reactor by scraping, and it was then diluted to 10^−5^ using sterile water and coated on a solid medium with PAM as the sole source of nitrogen. Screened pure strains of fungi were picked for spore inoculation into liquid medium that contained PAM for shake flask domestication. The inoculum of fungal spores was 15%. The liquid medium was composed of 400 mg PAM, 1000 mg glucose, 100 mg CaCl_2_, 500 mg MgSO_4_, 500 mg KH_2_PO_4_, and 20 mL soil (unfertilized garden) suspension (per L) (Chang et al., 2020). The culture period (25 ± 2°C, 150 rpm) lasted for 7 days, and after the formation of complete mycelial pellets, the pellets were collected and transferred to new culture flasks for further acclimation and expansion.

#### 2.1.3. Construction of the fungal-microalgal consortium

The optimal culture conditions for the most stable structure and the highest biomass in the shortest period were obtained after preliminary experiments. The consortium was constructed using the as-prepared synthetic wastewater in a flask culture system with 24 h light exposure (25 ± 2°C, 3,000 l×) and continual shaking at 150 rpm. The preliminary experiments revealed that the stability and biomass of the spheres formed by the inoculation of mycelia were higher than those formed by the inoculation of mycelial spheres that had already formed, and therefore, mycelia were inoculated into the microalgae to form the consortium used in this study. The fungal mycelia can potentially combine with the microalgae through rotation, entanglement, and adsorption to ultimately form a sphere, which resulted in a significant decrease in the microalgal biomass. Therefore, to ensure that the consortium can bind enough microalgal cells, the microalgae were first cultured to achieve a high OD value (OD_660_ = 0.8).

To establish whether the synergistic treatment effect of the microbial consortium on the wastewater that contained PAM was better than that of single fungal or microalgal systems, single system treatment experiments were conducted to compare the degradation by the three systems under the same culture conditions.

### 2.2. Composition of the synthetic wastewater

The synthetic PAM wastewater used in this study was composed of 400 mg PAM (anionic, molecular weight 3 × 10^7^), 1,000 mg NaHCO_3_, 100 mg CaCl_2_, 500 mg MgSO_4_, 500 mg KH_2_PO_4_, and 20 mL soil suspension (per L). A control experiment was performed to explore whether the inclusion of a co-metabolic nitrogen source could improve the degradation of PAM in which 750 mg/L urea, 60 mg/L NH_4_Cl or 300 mg/L yeast extract were added to the synthetic wastewater and compared to PAM as the sole nitrogen source.

### 2.3. Analytical methods

The concentration of PAM was determined using the starch cadmium iodide method (Zhao et al., 2019). A total organic carbon analyzer (TOC-VCPH, Shimadzu, Kyoto, Japan) was used to determine the concentrations of IC and TOC.

The removal efficiency (E, %) for the PAM, TOC and IC, which represents the percentage of pollutant mass removed from the influent, was calculated as follows:


$$E=\left[\left(C0-C1\right)/C0\right]\times 100\%$$

where *C*_0_ and *C*_1_ (mg/L) are the initial and end concentrations, respectively, in the solution.

Liquid samples of the PAM solution before and after sterilization and the HAPM solution after biodegradation were purified using methanol (Bao et al., 2010). The sample was added dropwise to a large excess of methanol, which caused the residual polymer in the sample to precipitate. The precipitated polymer was then filtered and washed twice with methanol before it was dried to a constant weight using a vacuum freeze dryer (SCIENTZ-18ND/A; Scientz Bio-Technology, Ningbo, China). After preparation, these samples were analyzed using Fourier transform infrared spectroscopy (FT-IR) (Thermo Nicolet, Thermo Fisher Scientific, Waltham, MA, United States).

Using a one-way analysis of variance (ANOVA), a statistical analysis was performed on the rates of degradation of PAM to determine significant differences (*p* < 0.05) among the three biological systems. The minimum significant difference between each pair of means was calculated.

The characterization was performed by analyzing the *18S rRNA* genes. The genomic DNA was extracted by collecting the cell precipitates from the original sample using a commercial DNA extraction kit (TGuide OSR-M502, TIANGEN Biotech, Beijing, China). The PCR was performed in a total volume of 50 μL that contained 25 μL polymerase premix (Takara Bio, Shiga, Japan), 1 μL DNA, 22 μL double-distilled H_2_O, and 1 μL each of the forward and reverse primers. The PCR amplification was conducted as follows: 95°C for 5 min, followed by 35 cycles of 45 s at 95°C, 45 s at 56°C, and 60 s at 72°C. The 18S rRNA gene was amplified with Fung and NS1 primers (Fung: 5′-ATTCCCCGTTACCCGTTG-3′; NS1: 5′-GTAGTCATATGCTTGTCTC-3′). The samples were sequenced using an ABI PRISM 3100 Genetic Analyzer (Applied Biosciences, Waltham, MA, United States). The sequenced fragments were analyzed by the program Staden Package 2.0.0b to obtain a consensus sequence. The consensus sequence was searched in GenBank using BLASTn. The sequence data of the strains obtained were uploaded to GenBank under the accession number OR426650.

## 3. Results and discussion

### 3.1. Isolation, identification and performance of the fungal strain used to degrade PAM

#### 3.1.1. Fungal isolation and identification

**Figure 1:**
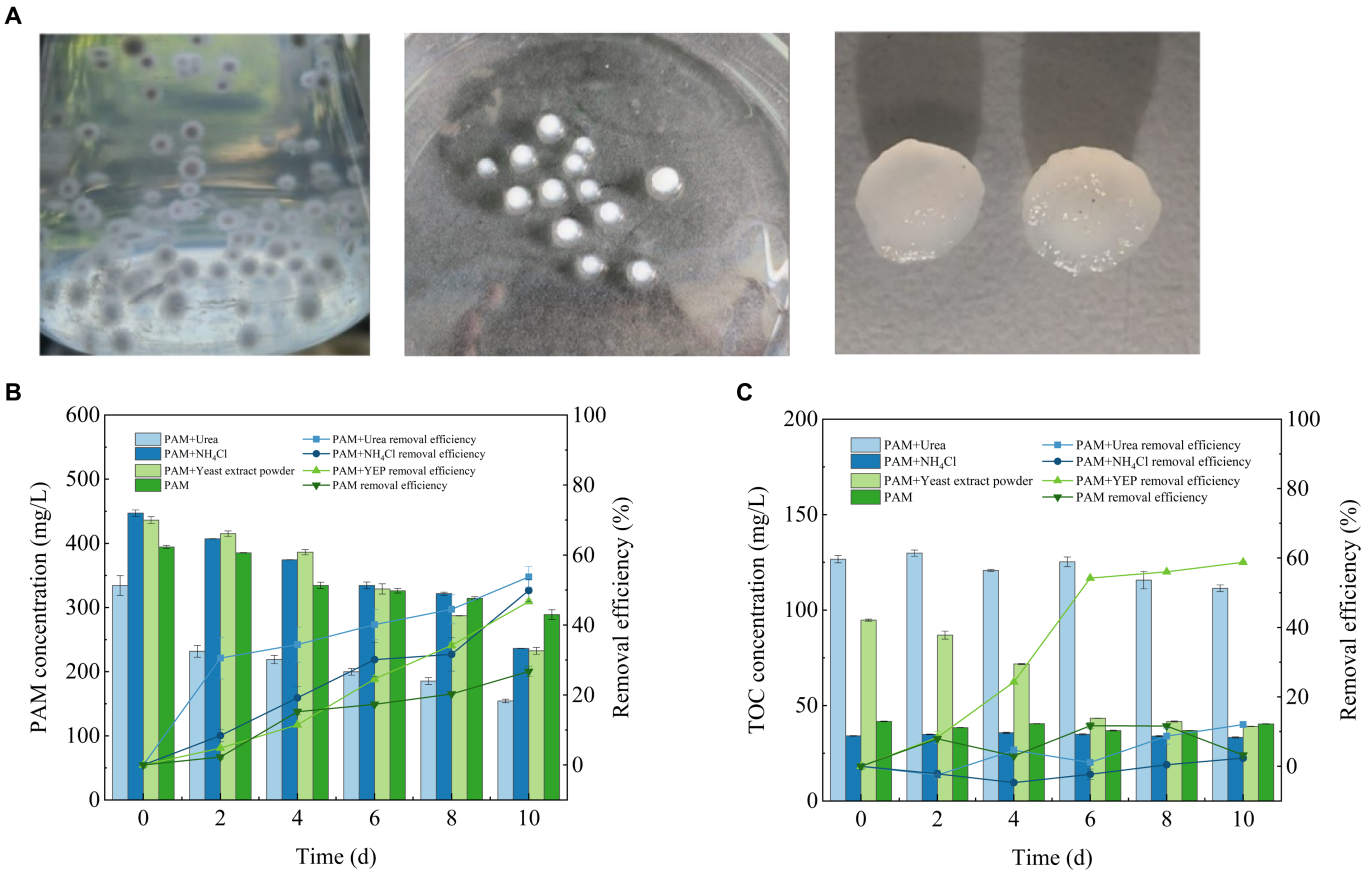
*Fusarium* mycelial pellet morphology **(A)** and its ability to remove PAM and TOC **(B)** PAM and **(C)** TOC. PAM, polyacrylamide; TOC, total organic carbon.

The pure strains were inoculated onto an inclined surface medium to detect the samples. After PCR amplification, the gene sequence that was isolated was identified as a species of *Fusarium* (Supplementary material). Fungi have mycelia, which use a process of winding and adsorption to form stable mycelial pellets in culture (Figure 1A). Compared with the use of single microbial strains to remove pollutants, the morphology and large specific surface area of the mycelial pellets enables greater contact and assimilation of the pollutants in wastewater (Li et al., 2023). More importantly, the formation of mycelial pellets is more conducive to the rapid separation of organisms and wastewater, which facilitates the reuse of microorganisms (Zhou et al., 2018). On this basis, the *Fusarium* mycelial pellet was used to package suspended algal cells, which consisted of the *Fusarium* mycelial pellet, and can effectively improve the ability to separate the algal cells and solution to achieve the goal of reusing them (Chen et al., 2020).

#### 3.1.2. Effect of the fungal mycelial in the synthetic flooding wastewater to degrade PAM

During isolation of the fungal strain, fungal growth was achieved on the plate when PAM was used as the sole nitrogen source, indicating that the isolated strain could effectively utilize PAM for its growth. The degradation of PAM was significantly affected by the addition of different co-metabolic nitrogen sources within 10 days of the completion of culture cycle (Figure 1B). The rate of degradation of PAM was the highest in the experimental group with added urea, which reached 53.76 ± 2.97%, and corresponded to a reduction in the concentration of PAM by approximately 200 mg/L. The rate of degradation of PAM reached 49.88 ± 3.2% in the experimental group with the addition of NH_4_Cl and 46.69 ± 0.57% with the addition of yeast extract, while only 26.74 ± 1.46% of the PAM was degraded when PAM was used as the sole nitrogen source.

A previous study showed that the process of PAM biodegradation begins with deamination by amidase catalysis (Nyyssölä and Ahlgren, 2019). As a polymeric compound, PAM cannot enter the intracellular portion of the *Fusarium* sp. cells through biofilms (Zhao et al., 2020). Therefore, when PAM is used as the sole source of nitrogen, *Fusarium* sp. cannot secrete many extracellular enzymes for a short time, thus, the efficiency of PAM degradation was the lowest. Furthermore, the addition of co-metabolic nitrogen sources could ensure the rapid growth of biomass, which results in the secretion of more extracellular amidase, thereby effectively improving the degradation of PAM. Therefore, the addition of a co-metabolic nitrogen source can promote the secretion of extracellular amidase, thereby improving the rate of degradation of PAM to some extent. The addition of urea promotes the secretion of extracellular amidase enzymes by *Fusarium* sp. and improves the rate of degradation of PAM, which is consistent with the findings of a previous study (Bao et al., 2010).

#### 3.1.3. Ability of the mycelial pellets to remove TOC in the synthetic flooding wastewater

During the culture period, the efficiency of TOC removal from the synthetic wastewater by *Fusarium* sp. exhibited significant differences when different co-metabolic nitrogen sources were added (Figure 1C). The concentration of TOC was significantly reduced in the experimental groups supplemented with yeast extract, which reached removal efficiencies of 58.77 ± 0.35%. The TOC removal efficiency in the other three groups was only approximately 10%. Compared with the other three nitrogen sources, yeast extract contains not only nitrogen but also many trace elements and growth factors, such as nucleosides and amino acids among others. It is hypothesized that these growth factors and trace elements are more conducive to the growth of fungi, which facilitates the synergistic degradation of PAM.

### 3.2. Performance of *Chlorella* sp. at treating synthetic flooding wastewater

#### 3.2.1. Growth curve of *Chlorella* sp. in the synthetic flooding wastewater

**Figure 2:**
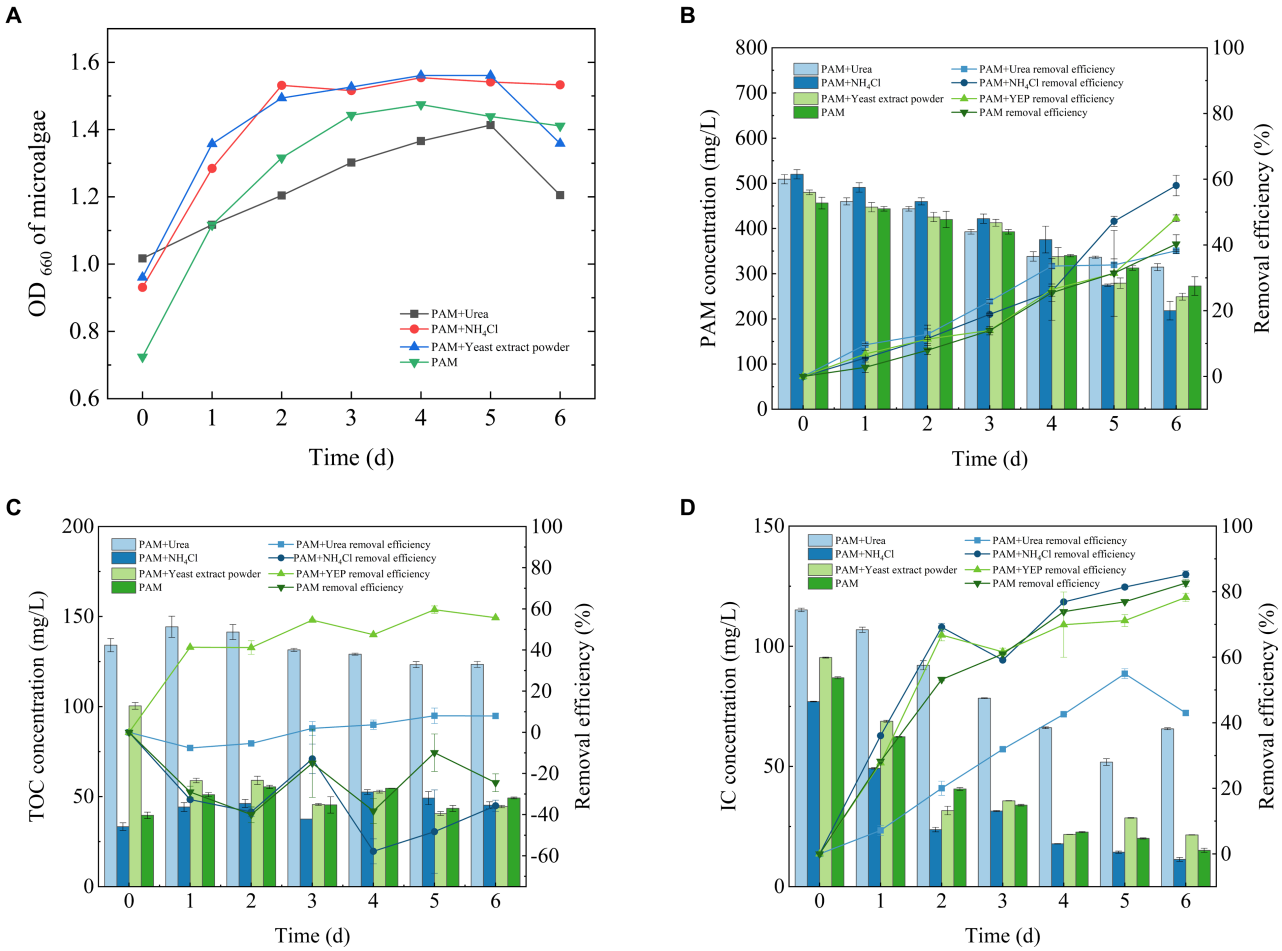
Growth curve of *Chlorella* sp. **(A)** and its ability to remove **(B)** PAM, **(C)** TOC and **(D)** IC. IC, inorganic carbon; PAM, polyacrylamide; TOC, total organic carbon.

The change in algal biomass during the 6 days incubation period is shown in Figure 2A. Algal cultures exhibited significant growth when different co-metabolic nitrogen sources were added and reached a stable biomass until approximately 5 days before they started to decline.

#### 3.2.2. Ability of *Chlorella* sp. to degrade PAM in the synthetic flooding wastewater

The degradation of PAM by *Chlorella* sp. was also affected by the addition of a co-metabolic nitrogen source (Figure 2B). The rate of degradation of PAM reached 58.07 ± 3.1% when supplemented with NH_4_Cl, 48.11 ± 1.05% when supplemented with yeast extract, 38.2 ± 0.27% when supplemented with urea and 40.28 ± 2.83% when PAM was used as the sole nitrogen source. To remove the organic nitrogen, algae first degraded it into ammonia using various enzymes, which enables it to then be absorbed by the algal cells (Simsek et al., 2016). These results indicate that the algae *Chlorella* sp. had a stronger ability to utilize inorganic nitrogen sources than those of organic nitrogen, although the degradation of PAM was effectively promoted by the addition of an external nitrogen source, regardless of whether it was organic or inorganic.

#### 3.2.3. Ability of *Chlorella* sp. to remove TOC and IC in the synthetic flooding wastewater

The ability of *Chlorella* sp. to remove TOC was significantly reduced during the 6 days culture cycle (Figure 2C). The rate of TOC removal reached 7.9 ± 1.28% when the system was supplemented with urea, −35.09 ± 2.72% when supplemented with NH_4_Cl, 55.08 ± 0.14% when supplemented with yeast extract, and −24.5 ± 4.32% when PAM was used as the sole nitrogen source. This result shows that the microalgae were more effective at degrading TOC when adequate nutrients were available. However, as the experimental period extended, the organic matter generated by microalgal physiological activity accumulated, which led to a potential decrease in the efficiency of TOC removal and an increase in the levels of TOC.

During the culture period, *Chlorella* sp. was highly efficient at removing the IC from wastewater that contained PAM (Figure 2D). Approximately 90% of the IC was removed, which confirmed the suitability of the system for carbon sequestration. The efficiency of the IC removal was 42.97 ± 0.74% when the system was supplemented with urea, 85.28 ± 1.05% when supplemented with NH_4_Cl, 79.14 ± 0.02% when supplemented with yeast extract and 82.62 ± 1.09% when PAM was used as the sole nitrogen source. This result also corresponds to a previous study that showed that microalgae are highly effective at fixing IC (Saravanan et al., 2022).

*Chlorella* can use both organic and IC sources (Nasir et al., 2015). A comparison of Figures 2C,D shows that when urea and yeast extract were used as co-metabolic nitrogen sources, the organic carbon in them could be utilized by *Chlorella*, thereby reducing the fixation of DIC in solution. Therefore, when urea and yeast extract were used as co-metabolic nitrogen sources, the TOC was removed when both nitrogen sources were used, but there was little removal of DIC. With ammonium chloride as a common metabolic nitrogen source and PAM as the only nitrogen source, there was little TOC in the system; *Chlorella* had to use DIC for growth, and during the process of growth, it secretes extracellular polymers, such as polysaccharides and proteins among others (Zhu et al., 2012). As a result, the two groups of TOC were negative, but the efficiency of the DIC fixed was positive.

### 3.3. Effectiveness of the *Fusarium*-*Chlorella* consortium at treating the synthetic flooding wastewater

#### 3.3.1. Morphology of the fungal-microalgal consortium pellets

**Figure 3:**
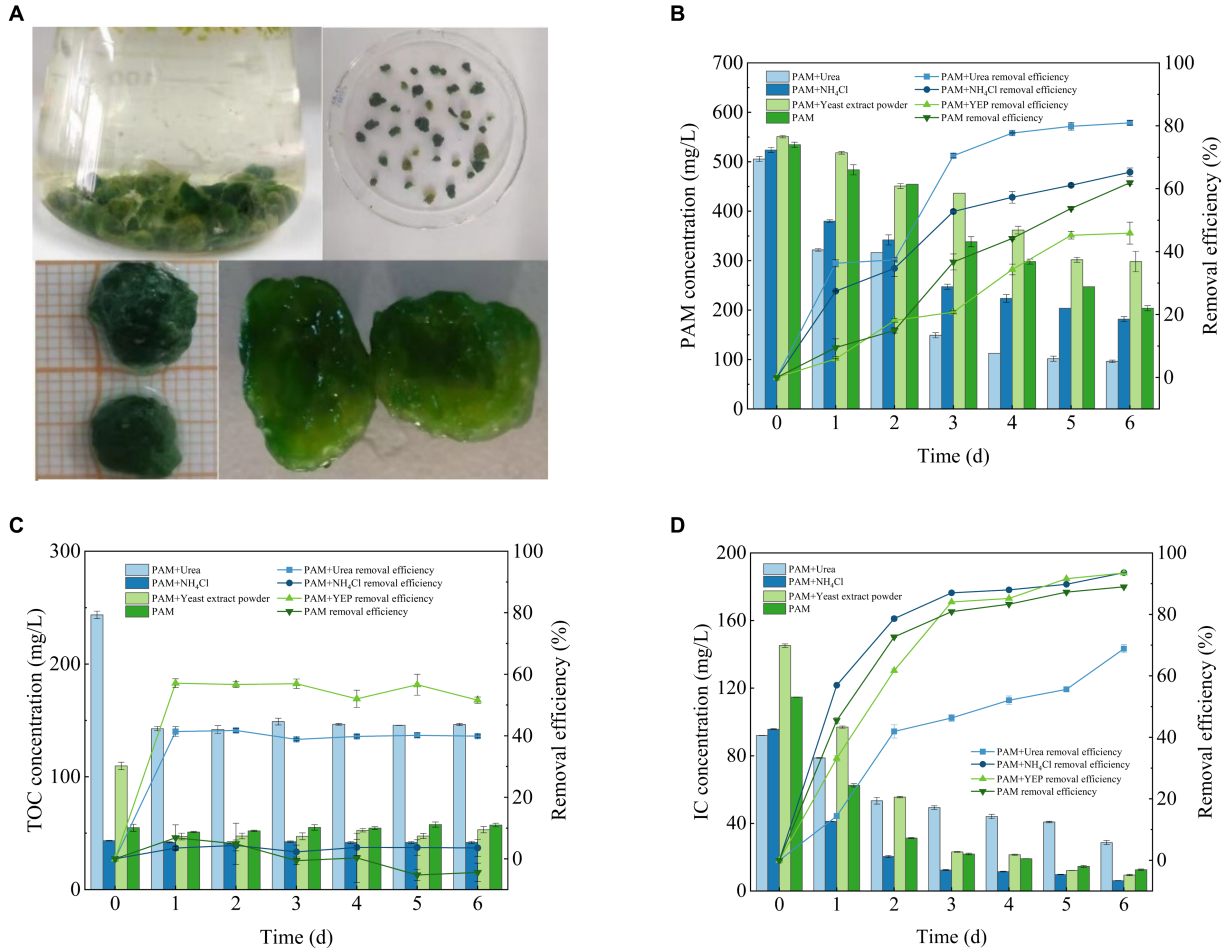
*Fusarium*-*Chlorella* consortium morphology **(A)** and its ability to remove **(B)** PAM, **(C)** TOC and **(D)** IC. IC, inorganic carbon; PAM, polyacrylamide; TOC, total organic carbon.

A previous study has described the mechanism of flocculation of the actinomycete *Streptomyces* sp. hsn06 on *Chlorella vulgaris* (Li et al., 2017) and demonstrated the potential ability of both microalgae and fungi to flocculate. Microalgae are mostly adsorbed onto the surface of mycelial pellets, while only a small number of microalgal cells exist within the internal structure of the fungal-microalgal consortium pellets (Figure 3A). This was consistent with the findings of previous research (Leng et al., 2021). The ratio of microalgae to fungi was 2:1. The final consortium sphere that was formed adsorbed a large number of microalgal cells with a dense and stable structure that exhibited excellent settling performance (Figure 3A). This is advantageous for the subsequent harvesting of the algal biomass. The consortium was subjected to stability experiments in a gas column with an aeration rate of 135 L/min, and the consortium could remain stable without disintegrating for 48 h.

#### 3.3.2. Ability of the *Fusarium*-*Chlorella* consortium to degrade PAM

The results described above showed that both algae and fungi could degrade PAM separately, and the performance of the *Fusarium*-*Chlorella* consortium at degrading PAM was higher than the performance of single systems (Figure 3B). The rate of degradation of PAM reached 80.93 ± 0.7% when the system was supplemented with urea, 65.27 ± 1.32% when supplemented with NH_4_Cl, 45.88 ± 3.48% when supplemented with yeast extract and 61.91 ± 0.6% when PAM was used as the sole nitrogen source. Overall, the concentration of PAM decreased significantly, and the rate of degradation reached 80%, which indicated that the *Fusarium*-*Chlorella* consortium had a significant effect on the degradation of PAM. Previous studies have shown that fungal-microalgal consortia have been widely used in wastewater treatment (Muradov et al., 2015), and the results of this study confirmed that the microbial consortium was highly adaptable to wastewater that contained PAM, which made it suitable for use in conditions where PAM is the sole nitrogen source available for growth.

#### 3.3.3. Ability of the *Fusarium*-*Chlorella* consortium to remove TOC and IC

The ability of the *Fusarium*-*Chlorella* consortium to remove TOC clearly differed when cultured with different co-metabolic nitrogen sources (Figure 3C). The rate of TOC removal reached 39.9 ± 0.41% when supplemented with urea, 3.56 ± 2.72% when supplemented with NH_4_Cl, 51.58 ± 1.05% when supplemented with yeast extract and −4.4 ± 3.0% when PAM was used as the sole source of nitrogen. Therefore, the experimental groups supplemented with organic nitrogen sources provided another source of TOC in addition to PAM, which resulted in a significant decrease in the concentration of TOC. This may be due to the full utilization of additional sources of organic nitrogen. However, in the experimental groups where the inorganic nitrogen sources were supplemented or PAM was used as the sole nitrogen source, the concentration of TOC remained relatively stable with no significant changes observed. This may be attributed to the accumulation of some organic compounds that resulted from microbial metabolism during the cultivation period that were not utilized.

The degradation of IC by the *Fusarium*-*Chlorella* consortium over the 6 days culture period is shown in Figure 3D. The rate of IC removal reached 68.83 ± 1.32% in the group supplemented with urea, 93.58 ± 0.06% when supplemented with NH_4_Cl, 93.48 ± 0.27% when supplemented with yeast extract and 88.97 ± 0.36% when PAM was used as the sole nitrogen source. Overall, the synergistic performance of the *Fusarium*-*Chlorella* consortium to treat IC was better than that of the single *Chlorella* sp. system with the ability to sequester carbon providing obvious advantages. These findings prove that the mechanisms of biodegradation by microalgae and fungi have synergistic effects, which results in their effective combination for wastewater treatment (Wang et al., 2022).

An analysis of the differences in the degradation efficiency of PAM among three different biological systems that did not include co-metabolic nitrogen sources was conducted. The results demonstrated a significant difference (*p* = 0.003) in the degradation capability among the three systems. This indicates that different biological systems exhibit significantly different abilities to degrade PAM, and the efficiency of the consortium to degrade PAM is significantly higher than that of a single system.

According to China’s Petrochemical Pollutant Discharge Standard (GB31570-2015, China), the concentrations of PAM and IC in effluent are not clearly required, while the relevant stipulated parameter is the chemical oxygen demand (COD). In the GB31570-2015, the COD discharge standard in oil flooding wastewater requires less than 100 mg/L. The COD values of the simulated flooding wastewater treated by mycelia were usually between 150 mg/L and 200 mg/L, which cannot directly reach the standard. However, in practical applications, oil flooding wastewater is treated by combining a variety of treatment processes, such as anaerobic baffled treatment process and aerobic biofilm treatment process among others (Sang et al., 2015; Song et al., 2018, 2019). In practice, the mycelial pellet can be combined with various processes to successfully process the DIC.

### 3.4. Mechanisms for the removal of pollutants

#### 3.4.1. Functional group analysis of PAM after biodegradation

**Figure 4:**
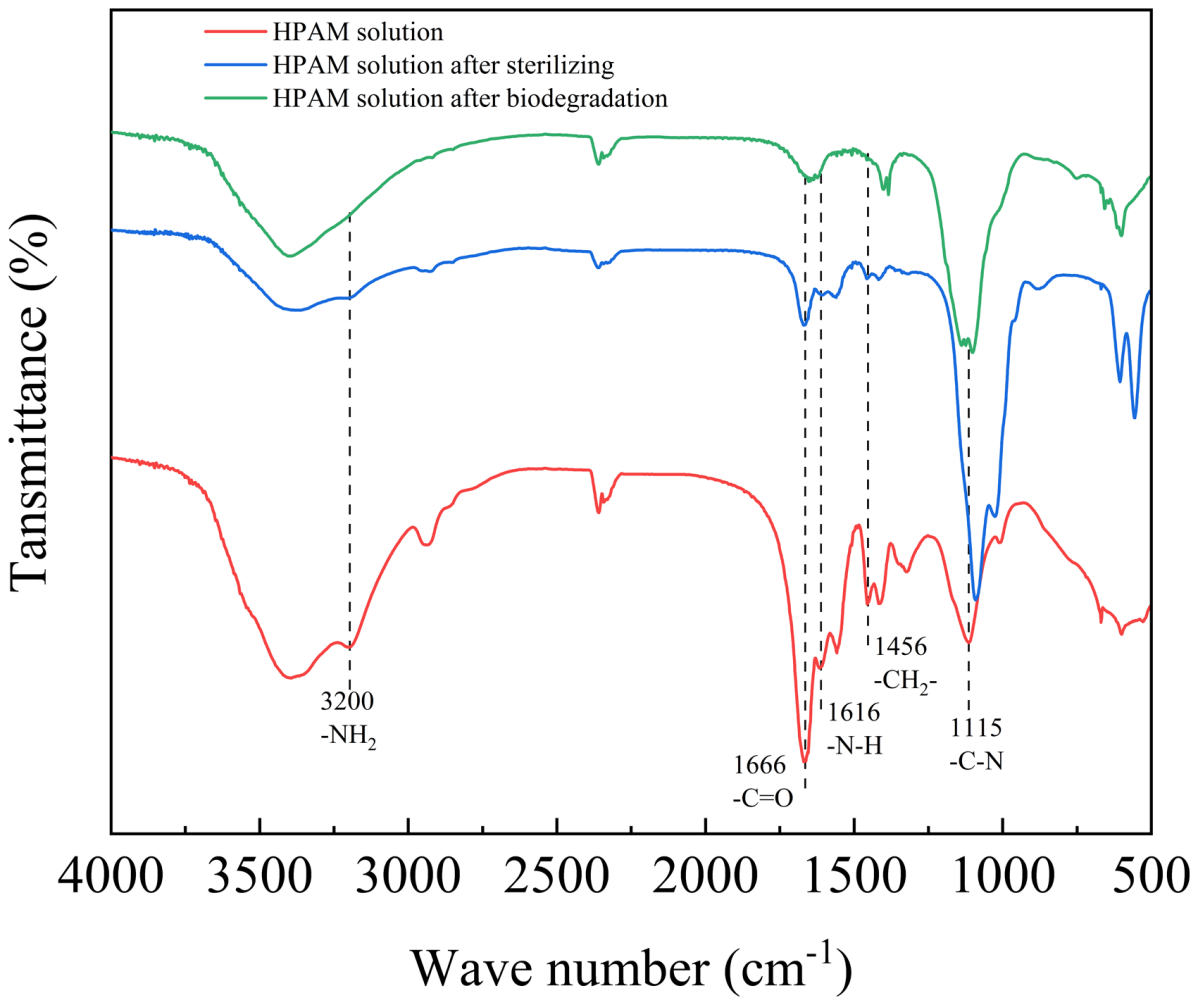
FT-IR analysis of PAM before and after biodegradation by the *Fusarium*-*Chlorella* consortium. FT-IR, Fourier transform infrared spectroscopy; PAM, polyacrylamide.

To investigate the structural changes to PAM molecules after sterilization and biodegradation, the FT-IR spectra of different PAM samples were analyzed (Figure 4). The peaks observed at 1115, 1616, 1,666 and 3,200 cm^−1^ in the spectrum of PAM corresponded to the existence of C–N, N–H, C=O bonds and amide groups (Bao et al., 2010; Zhao et al., 2019).

A comparison of the FT-IR spectra for all three samples showed that the peaks that represented the C–N bonds, N–H bonds, C=O bonds and -NH_2_ gradually disappeared, indicating that the side chains and amide groups of PAM were degraded. Furthermore, the peak that represented methylene at 1456 cm^−1^ also gradually disappeared, indicating that the carbon backbone of PAM was also degraded. Furthermore, upon comparing the spectra of the three samples between 3,100 cm^−1^ and 3,500 cm^−1^, it was observed that the peaks had become wider. This indicated that the amide group had degraded into a carboxyl group (Bao et al., 2010). In comparison to the PAM degradation effects of high temperature sterilization and biodegradation, it was apparent that high temperature sterilization has some effect at degrading PAM, and the subsequent biodegradation fully degraded the PAM.

#### 3.4.2. Possible mechanisms for the removal of PAM and IC

Based on the studies described above, three possible mechanisms for the removal of PAM, as well as that of the IC, by the biological systems were initially proposed.

When the experimental group without the use of additional nitrogen sources was used as an example, according to the metabolic pathway proposed in a previous study (Zhao et al., 2019) and the FT-IR results described above, the side chains of PAM were cleaved, which may be due to the role of extracellular amidases secreted by the microalgae and fungi to convert the amide group to carboxylate group; however, there was no carboxylate group that bound intramolecularly, which was slightly different from the findings of previous studies (Sang et al., 2015). Alternatively, it also proved that the microorganisms in this study could grow using PAM as the sole nitrogen source. In addition, the biomass still grew without the addition of an organic nitrogen source, and the FT-IR results showed that the main carbon chain of PAM was cleaved, which might be the result of the catalytic degradation of this carbon chain of PAM by a monooxygenase secreted by the microorganisms (Zhao et al., 2019). It also showed that PAM can be used as the sole source of carbon for microbial growth.

To remove the IC, we primarily rely on the biological fixation of IC by the microalgae in soluble carbonate compounds, such as NaHCO_3_ (Molazadeh et al., 2019). Research has shown that microalgae primarily use CA to metabolize IC (Thanigaivel et al., 2022). In the Calvin cycle, microalgae absorb CO_2_ and synthesize organic compounds to fix CO_2_, and when the CO_2_ dissolves, it converts the carbon into various forms of IC, thereby enabling the microalgae to remove the IC.

## 4. Conclusion

The assisted flocculation of the fungal spores can effectively immobilize the microalgal cells and achieve a stable consortium in a short culture cycle. The removal effect of the *Fusarium*-*Chlorella* consortium for PAM and IC was much better than that of the two separate culture systems, and the efficiency of degradation of the pollutants could be significantly improved by the addition of an additional nitrogen source. The effluent of the degraded water was free of toxic byproducts, and the whole process was inexpensive and highly efficient, and it also provided ideas for the effective recovery of microalgae.

## Data availability statement

The datasets presented in this study can be found in online repositories. The names of the repository/repositories and accession number(s) can be found below: GenBank, OR426650.

## Author contributions

HZ: Funding acquisition, Writing – original draft. MS: Formal analysis, Investigation, Writing – original draft. CZ: —. ZP: —. ZA: Funding acquisition, Writing – review & editing.

## References

[ref1] Albalawi A. Alhasani R. H. A. Biswas L. Reilly J. Akhtar S. Shu X. (2018). Carnosic acid attenuates acrylamide-induced retinal toxicity in zebrafish embryos. Exp. Eye Res. 175, 103–114. doi: 10.1016/j.exer.2018.06.018, PMID: 29928899

[ref2] Bao M. Chen Q. Li Y. Jiang G. (2010). Biodegradation of partially hydrolyzed polyacrylamide by bacteria isolated from production water after polymer flooding in an oil field. J. Hazard. Mater. 184, 105–110. doi: 10.1016/j.jhazmat.2010.08.011, PMID: 20813455

[ref3] Chang C. Zhang J. Liu T. Song K. Xie J. Luo S.. (2020). Rhizosphere fungal communities of wild and cultivated soybeans grown in three different soil suspensions. Appl. Soil Ecol. 153:103586. doi: 10.1016/j.apsoil.2020.103586

[ref4] Chen J. Ding L. Liu R. Xu S. Li L. Gao L.. (2020). Hydrothermal carbonization of microalgae-fungal pellets: removal of nutrients from the aqueous phase fungi and microalgae cultivation. ACS Sustain. Chem. Eng. 8, 16823–16832. doi: 10.1021/acssuschemeng.0c05441

[ref5] Du Z. Y. Alvaro J. Hyden B. Zienkiewicz K. Benning N. Zienkiewicz A.. (2018). Enhancing oil production and harvest by combining the marine alga *Nannochloropsis oceanica* and the oleaginous fungus *Mortierella elongata*. Biotechnol. Biofuels 11:174. doi: 10.1186/s13068-018-1172-2, PMID: 29977335 PMC6013958

[ref6] Giménez-Gómez P. Hättestrand I. Sjöberg S. Dupraz C. Richardson S. Pamme N. (2023). Distance-based paper analytical device for the determination of dissolved inorganic carbon concentration in freshwater. Sens. Actuators B 385:133694. doi: 10.1016/j.snb.2023.133694

[ref7] Leng L. Li W. Chen J. Leng S. Chen J. Wei L.. (2021). Co-culture of fungi-microalgae consortium for wastewater treatment: a review. Bioresour. Technol. 330:125008. doi: 10.1016/j.biortech.2021.125008, PMID: 33773267

[ref8] Li L. Liang T. Qiu S. Zhang Y. Qu J. Liu T.. (2023). A rapid and simplified method for evaluating the performance of fungi-algae pellets: a hierarchical analysis model. Sci. Total Environ. 860:160442. doi: 10.1016/j.scitotenv.2022.160442, PMID: 36435261

[ref9] Li Y. Xu Y. Zheng T. Wang H. (2017). Flocculation mechanism of the actinomycete Streptomyces sp. hsn06 on *Chlorella vulgaris*. Bioresour. Technol. 239, 137–143. doi: 10.1016/j.biortech.2017.05.028, PMID: 28521222

[ref10] Liu X. Fu Q. Liu Z. Zeng T. Du M. He D.. (2021). Alkaline pre-fermentation for anaerobic digestion of polyacrylamide flocculated sludge: simultaneously enhancing methane production and polyacrylamide degradation. Chem. Eng. J. 425:131407. doi: 10.1016/j.cej.2021.131407

[ref11] Ma L. Hu T. Liu Y. Liu J. Wang Y. Wang P.. (2021). Combination of biochar and immobilized bacteria accelerates polyacrylamide biodegradation in soil by both bio-augmentation and bio-stimulation strategies. J. Hazard. Mater. 405:124086. doi: 10.1016/j.jhazmat.2020.124086, PMID: 33153796

[ref12] Ma Y. Yu H. Pan W. Liu C. Zhang S. Shen Z. (2010). Identification of nitrile hydratase-producing *Rhodococcus ruber* TH and characterization of an amiE-negative mutant. Bioresour. Technol. 101, 285–291. doi: 10.1016/j.biortech.2009.07.057, PMID: 19720524

[ref13] Modak N. M. Ghosh D. K. Panda S. Sana S. S. (2018). Managing green house gas emission cost and pricing policies in a two-echelon supply chain. CIRP. J. Manuf. Sci. Technol. 20, 1–11. doi: 10.1016/j.cirpj.2017.08.001

[ref14] Molazadeh M. Ahmadzadeh H. Pourianfar H. R. Lyon S. Rampelotto P. H. (2019). The use of microalgae for coupling wastewater treatment with CO_2_ biofixation. Front. Bioeng. Biotechnol. 7:42. doi: 10.3389/fbioe.2019.00042, PMID: 30941348 PMC6433782

[ref15] Muradov N. Taha M. Miranda A. F. Wrede D. Kadali K. Gujar A.. (2015). Fungal-assisted algal flocculation: application in wastewater treatment and biofuel production. Biotechnol. Biofuels 8:24. doi: 10.1186/s13068-015-0210-6, PMID: 25763102 PMC4355497

[ref16] Nasir N. M. Bakar N. S. A. Lananan F. Abdul Hamid S. H. Lam S. S. Jusoh A. (2015). Treatment of African catfish, *Clarias gariepinus* wastewater utilizing phytoremediation of microalgae, *Chlorella* sp. with *Aspergillus niger* bio-harvesting. Bioresour. Technol. 190, 492–498. doi: 10.1016/j.biortech.2015.03.023, PMID: 25791330

[ref17] Nyyssölä A. Ahlgren J. (2019). Microbial degradation of polyacrylamide and the deamination product polyacrylate. Int. Bioteder. Biodegrad. 139, 24–33. doi: 10.1016/j.ibiod.2019.02.005

[ref18] Sang G. Pi Y. Bao M. Li Y. Lu J. (2015). Biodegradation for hydrolyzed polyacrylamide in the anaerobic baffled reactor combined aeration tank. Ecol. Eng. 84, 121–127. doi: 10.1016/j.ecoleng.2015.07.028

[ref19] Saravanan A. Deivayanai V. C. Senthil Kumar P. Rangasamy G. Varjani S. (2022). CO_2_ bio-mitigation using genetically modified algae and biofuel production towards a carbon net-zero society. Bioresour. Technol. 363:127982. doi: 10.1016/j.biortech.2022.127982, PMID: 36126842

[ref20] Saravanan A. Karishma S. Kumar P. S. Rangasamy G. (2023). Biodegradation of oil-contaminated aqueous ecosystem using an immobilized fungi biomass and kinetic study. Environ. Res. 220:115252. doi: 10.1016/j.envres.2023.115252, PMID: 36632883

[ref21] Sheng Y. Benmati M. Guendouzi S. Benmati H. Yuan Y. Song J.. (2022). Latest eco-friendly approaches for pesticides decontamination using microorganisms and consortia microalgae: a comprehensive insights, challenges, and perspectives. Chemosphere 308:136183. doi: 10.1016/j.chemosphere.2022.136183, PMID: 36058371

[ref22] Simsek H. Kasi M. Ohm J.-B. Murthy S. Khan E. (2016). Impact of solids retention time on dissolved organic nitrogen and its biodegradability in treated wastewater. Water Res. 92, 44–51. doi: 10.1016/j.watres.2016.01.041, PMID: 26841227

[ref23] Song T. Li S. Ding W. Li H. Bao M. Li Y. (2018). Biodegradation of hydrolyzed polyacrylamide by the combined expanded granular sludge bed reactor-aerobic biofilm reactor biosystem and key microorganisms involved in this bioprocess. Bioresour. Technol. 263, 153–162. doi: 10.1016/j.biortech.2018.04.121, PMID: 29738978

[ref24] Song T. Li S. Jin J. Yin Z. Lu Y. Bao M.. (2019). Enhanced hydrolyzed polyacrylamide removal from water by an aerobic biofilm reactor-ozone reactor-aerobic biofilm reactor hybrid treatment system: performance, key enzymes and functional microorganisms. Bioresour. Technol. 291:121811. doi: 10.1016/j.biortech.2019.121811, PMID: 31344634

[ref25] Song T. W. Li S. S. Yin Z. C. Bao M. T. Lu J. R. Li Y. (2021). Hydrolyzed polyacrylamide-containing wastewater treatment using ozone reactor-up flow anaerobic sludge blanket reactor-aerobic biofilm reactor multistage treatment system. Environ. Pollut. 269:116111. doi: 10.1016/j.envpol.2020.116111, PMID: 33290953

[ref26] Thanigaivel S. Vickram S. Manikandan S. Deena S. R. Subbaiya R. Karmegam N.. (2022). Sustainability and carbon neutralization trends in microalgae bioenergy production from wastewater treatment: a review. Bioresour. Technol. 364:128057. doi: 10.1016/j.biortech.2022.128057, PMID: 36195218

[ref27] Vyrides I. Andronikou M. Kyprianou A. Modic A. Filippeti A. Yiakoumis C.. (2018). CO_2_ conversion to CH_4_ using zero valent iron (ZVI) and anaerobic granular sludge: optimum batch conditions and microbial pathways. J. CO_2_ Util. 27, 415–422. doi: 10.1016/j.jcou.2018.08.023

[ref28] Wang S.-K. Yang K.-X. Zhu Y.-R. Zhu X.-Y. Nie D.-F. Jiao N.. (2022). One-step co-cultivation and flocculation of microalgae with filamentous fungi to valorize starch wastewater into high-value biomass. Bioresour. Technol. 361:127625. doi: 10.1016/j.biortech.2022.127625, PMID: 35850393

[ref29] Zain Ul Arifeen M. Ma Y. Wu T. Chu C. Liu X. Jiang J.. (2022). Anaerobic biodegradation of polycyclic aromatic hydrocarbons (PAHs) by fungi isolated from anaerobic coal-associated sediments at 2.5 km below the seafloor. Chemosphere 303:135062. doi: 10.1016/j.chemosphere.2022.135062, PMID: 35618067

[ref30] Zhang H. Li X. An Z. Liu Z. Tang C. Zhao X. (2021). Treatment of polyacrylamide-polluted wastewater using a revolving algae biofilm reactor: pollutant removal performance and microbial community characterization. Bioresour. Technol. 332:125132. doi: 10.1016/j.biortech.2021.125132, PMID: 33848818

[ref31] Zhao L. Song T. Han D. Bao M. Lu J. (2019). Hydrolyzed polyacrylamide biotransformation in an up-flow anaerobic sludge blanket reactor system: key enzymes, functional microorganisms, and biodegradation mechanisms. Bioprocess Biosyst. Eng. 42, 941–951. doi: 10.1007/s00449-019-02094-w, PMID: 30820666

[ref32] Zhao L. Zhang C. Lu Z. Bao M. Lu J. (2020). Key role of different levels of dissolved oxygen in hydrolyzed polyacrylamide bioconversion: focusing on metabolic products, key enzymes and functional microorganisms. Bioresour. Technol. 306:123089. doi: 10.1016/j.biortech.2020.123089, PMID: 32155564

[ref33] Zhou K. Zhang Y. Jia X. (2018). Co-cultivation of fungal-microalgal strains in biogas slurry and biogas purification under different initial CO_2_ concentrations. Sci. Rep. 8:7786. doi: 10.1038/s41598-018-26141-w 29773893 PMC5958114

[ref34] Zhu L. Qi H. Lv M. Kong Y. Yu Y. Xu X. (2012). Component analysis of extracellular polymeric substances (EPS) during aerobic sludge granulation using FTIR and 3D-EEM technologies. Bioresour. Technol. 124, 455–459. doi: 10.1016/j.biortech.2012.08.059, PMID: 23022627

