# Supplementary figures and images for: Construction of a mycelium sphere using a Fusarium strain isolate and Chlorella sp. for polyacrylamide biodegradation and inorganic carbon fixation
Source: Front Microbiol. 2023 Oct 5;14:1270658. doi: 10.3389/fmicb.2023.1270658 (PMC10585063; doi:10.3389/fmicb.2023.1270658)

## *Supplementary Material*

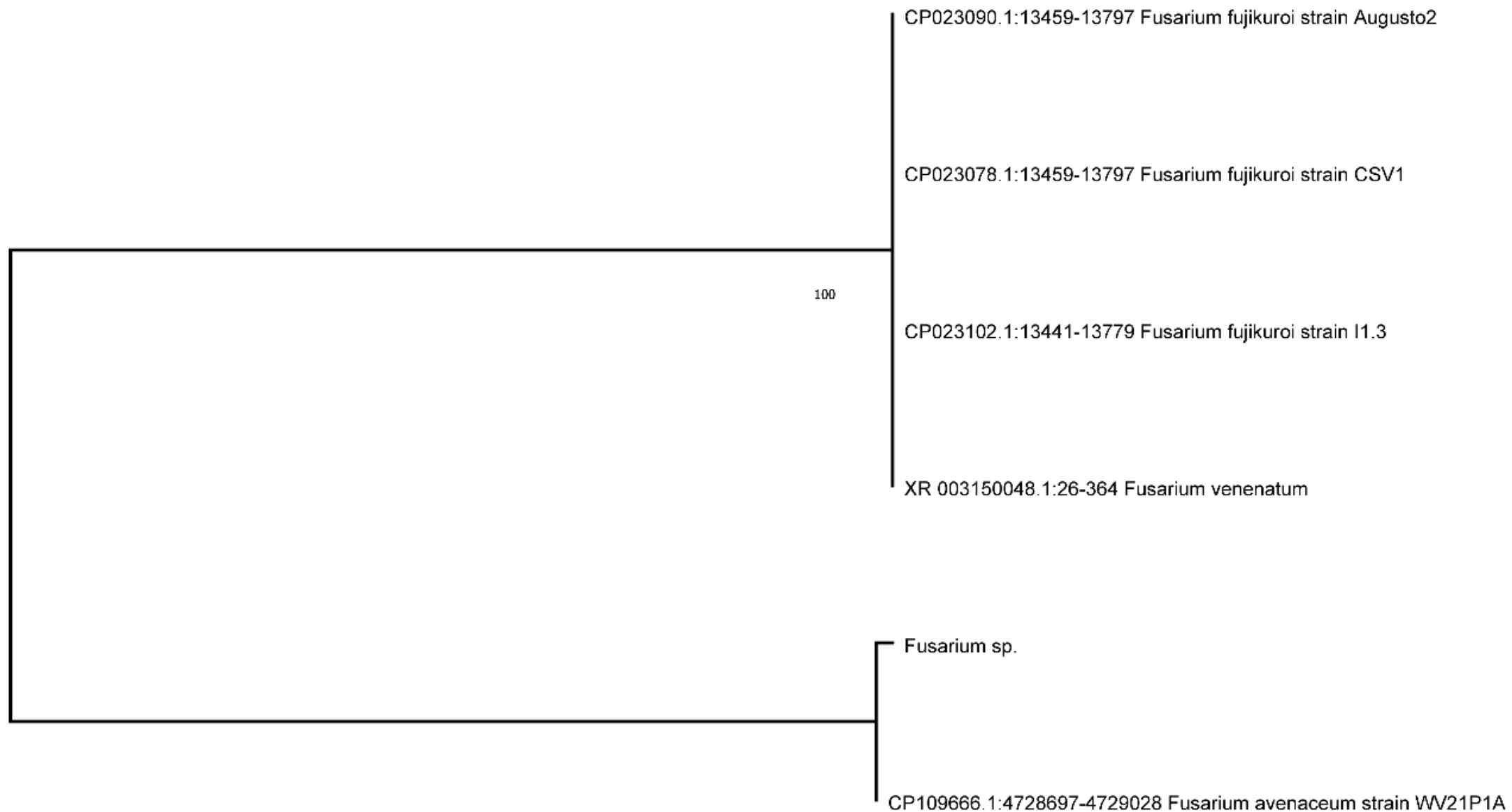

**Supplementary Figure 1.** Phylogenetic tree of *Fusarium* sp.

Supplement: Supplementary file 1 [file Image_1.pdf]
